# Finite Element Analysis of a New Type of Spinal Protection Device for the Prevention and Treatment of Osteoporotic Vertebral Compression Fractures

**DOI:** 10.1111/os.13220

**Published:** 2022-02-11

**Authors:** Mingxue Che, Yongjie Wang, Yao Zhao, Shaokun Zhang, Jun Yu, Weiquan Gong, Debao Zhang, Mingxi Liu

**Affiliations:** ^1^ Department of Spinal Surgery The First Hospital of Jilin University Changchun China; ^2^ Jilin Engineering Research Center for Spine and Spinal Cord Injury Changchun China; ^3^ Department of Joint Surgery The First Hospital of Jilin University Changchun China; ^4^ Department of medical imaging Jilin Provincial Armed Police Corps Hospital Changchun China; ^5^ Department of Orthopaedic Traumatology The First Hospital of Jilin University Changchun China

**Keywords:** Finite element analysis, Osteoporosis, Stress distribution, Osteoporotic vertebral fracture, Spinal protection

## Abstract

**Objective:**

To study the effectiveness of a new spinal protection device for preventing and treating osteoporotic vertebral compression fractures (OVCFs) by finite element analysis (FEA).

**Methods:**

One healthy volunteer and one patient with 1‐segment lumbar vertebral compression fractures were included in this experimental study. The DICOM files of two different lumbar spiral computed tomography (CT) scans were converted into STL files, and 3D finite element models of the lumbar spine were generated for normal and L1 vertebral fracture spines. A new type of spinal protection device was applied to reduce the stress on the anterior vertebral edge and direct the center of gravity posteriorly. The stress distribution characteristics of different finite element models of the lumbar spine were analyzed, revealing the characteristics of the stress distributed along the spine under the action of the new spinal protection device.

**Results:**

Under normal conditions, the stress was mainly distributed in the middle and posterior columns of the spine. When the anterior border of the L1 vertebral body was fractured and collapsed, the stress distribution shifted toward the anterior column due to the center of gravity being directed forward. According to finite element analysis of the spine with the new protection device, the stress in the middle and posterior columns tended to increase, and that in the anterior column decreased. After the new type of spinal fixation device was applied, the stress at the L1 and L2 vertebral endplates decreased to a certain extent, especially that at the L1 vertebral body. The maximum stress on the L1 vertebral body decreased by 20% after the auxiliary device was applied.

**Conclusions:**

According to the FEA results, the new spinal protection device can effectively prevent and treat osteoporotic vertebral compression fractures (OVCFs), and can alter the stress distribution in the spine and reduce the stress in the anterior column of the vertebral body, especially in vertebral compression fractures.

## Graphical abstract

We found that when the anterior border of the L1 vertebral body was fractured and collapsed, the stress distribution shifted toward the anterior column due to the change of the center of gravity. According to finite element analysis of the spine with the new protection device, the stress in the middle and posterior columns tended to increase, and that in the anterior column decreased, indicating that this device can effectively alter the stress distribution in the spine and treat OVCFs.

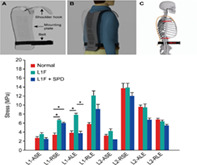

## Introduction

With the aging of society, the occurrence of osteoporotic vertebral compression fractures (OVCFs) is increasing, especially in the elderly population, causing acute or chronic pain episodes, progressive spinal deformities and increasing the financial burden of both patients and society. As population aging progresses over time, the elderly population will account for one‐third of the population by ~2050. Consequently, the incidence rate of osteoporosis (OP) will increase, and the incidence of OVCFs will also become higher and higher. Osteoporosis is characterized by the loss of mass and damage to the bone microstructure, leading to increased brittleness, generalized bone disorders, and a high risk of fracture.^1^ The imbalance between osteoblasts and osteoclasts leads to a negative balance between bone resorption and bone remodeling.^2^ Vertebral compression fractures due to osteoporosis occurred frequently. The main symptom of osteoporotic vertebral compression fractures is low back pain, which is rarely addressed early. It was speculated that the changes in the stress distribution caused by the forward‐directed center of gravity after the occurrence of a compression fracture of the vertebral body are the main causes of the recurrence of vertebral fractures within a short period of time after OVCFs. Patients who have a history of a segmental vertebral fracture have a 5‐fold increased risk of incurring another vertebral fracture, regardless of their bone density. Moreover, those who have a history of two or more vertebral fractures have a 12‐fold increased risk of incurring another vertebral fracture. For patients with a history of vertebral fracture, subsequent fractures are more severe.^3^, ^4^ Osteoporotic vertebral compression fractures can cause intractable low back pain, movement disorders, spinal deformities and even disabilities, seriously affecting the quality of life of elderly people.^5^, ^6^, ^7^, ^8^

Currently, there are no clear criteria and no effective approaches that can prevent and treat spinal fractures. Treatments for OVCFs mainly include bed rest, traditional Chinese medicine functional reduction, percutaneous kyphoplasty (PKP), percutaneous vertebroplasty (PVP), etc., which cannot solve the problem of long‐term bed rest or re‐fracture. To date, PVP and PKP are universally known as appropriate vertebral augmentation procedures for OVCFs because they have many advantages, such as short surgical time, no general anesthesia, and quick pain relief. However, loss of vertebral height, cement leakage, and adjacent vertebral refracture are still unsolved problems of these approaches. A large number of patients with osteoporotic vertebral compression fractures may need surgical intervention. However, surgical intervention brings substantial economic burden to both individuals and society.^9^ Therefore, it is necessary to develop effective prevention and treatment strategies for patients with OVCFs and high‐risk groups to avoid or slow the occurrence of fractures.

Finite element analysis (FEA) has been widely used in spine biomechanics research, such as stress analysis after spinal internal fixation and development of spinal molds. FEA can detect the internal interaction mechanism of each segment of human body, and effectively analyze the biomechanical changes of osteoporotic vertebral fracture, which may become the gold standard of bone strength research.^10^ With finite element modeling, the mechanical environment of OVCFs in the spine can be simulated, the fracture mechanism can be identified, and the hypotheses can be tested. Therefore, new interventions can be tested by FEA for its effectiveness for the prevention and treatment of OVCFs.

The arrangement of the thoracic and lumbar segments (T11‐L2) of the spinal articular processes is different in the coronal plane and sagittal plane. Under external forces, the stiffness of the vertebral body increases sharply, resulting in stress concentration and high fracture risks. OVCF is the most common type, accounting for ~80% of fractures. In this research, we hypothesized that the new type of spinal protection device can help to stabilize the spine, reduce the stress, and prevent the occurrence of OVCFs and re‐fracture. Therefore, the objective of the current study was to: (i) create a finite element model to observe the stress distribution characteristics of L1 vertebral body and analyze the impact of compression fractures on the stress distribution; (ii) design a new type of spinal protection device according to the biomechanical characteristics of the spine to stabilize the spine, reduce the stress, and prevent the occurrence of OVCF and re‐fracture; and (iii) analyze the influence of this new spinal protection device on the stress distribution in OVCF spine mechanics through a finite element model, and determine the prevention and treatment effects of this device for OVCFs and vertebral re‐fracture.

## Materials and methods

### Inclusion criteria

The inclusion criteria were as follows: (i) patients aged 40–60 years; (ii) patients with or without 1‐segment lumbar vertebral compression fractures; (iii) osteoporosis; and (iv) no surgical treatment was performed.

### Exclusion criteria

The exclusion criteria were as follows: (i) underwent anti‐osteoporosis treatment before OVCF; (ii) have a history of spinal surgery; (iii) have diseases influencing bone resorption and remodeling other than osteoporosis; and (iv) have a period of follow‐up <6 months.

### Patients

From January 2020 to May 2020, the medical records for patients with osteoporosis in the First Hospital of Jilin University were retrospectively reviewed. After applying the inclusion and exclusion criteria, we selected one healthy volunteer (woman, 47 years) and one patient (man, 58 years) with 1‐segment lumbar vertebral compression fracture in this study. Ismail et al.^11^ divided OVCFs into three categories according to whether there was a wedge, double concavity, and collapsing to study the relationship between the number and type of vertebral malformations and low back pain and height loss. In this study, only the data of patients with mild lumbar deformities with a 20% reduction in the height of the anterior vertebral body were selected for FEA.

### Treatment

The two participants both received X‐rays to rule out thoracolumbar deformities. Then, 64‐row spiral CT scans from T12 to L5 were performed with a GE Light Speed scanner (General Electric Company, Boston, USA), and the files were saved in the DICOM format for following establishment of 3D finite element model of L1 vertebral body with or without compression fractures. After the new type of spinal protection device was applied according to the biomechanical characteristics of the spine, a finite element model for the spinal protection device was established for following analysis.

### Outcome measures

#### Vertebral fracture assessment: semiquantitative technique

A semiquantitative method was used to grade the fractured vertebral body (grade 0–3): normal vertebral body height (grade 0), mild deformity (grade 1, ~20%–25% reduction in front/back comparison), moderate deformity (grade 2, ~25%–40% reduction in arbitrary height, ~20%–40% reduction in area), and severe deformity (grade 3, ~40% reduction in any height or area).^12^

#### Load and boundary condition assessment

According to the three‐column concept of the spine, loads and torques are applied to the upper endplate and articular surface of the T12 vertebral body, 85% of which are located in the anterior and middle column and 15% in the posterior column.^13^, ^14^ The pre‐test results showed that the most concentrated area of stress on the lumbar spine was on the middle column, so the anterior and middle columns of the lumbar model were used as equivalent stress sampling areas in this test.

**Fig. 1:**
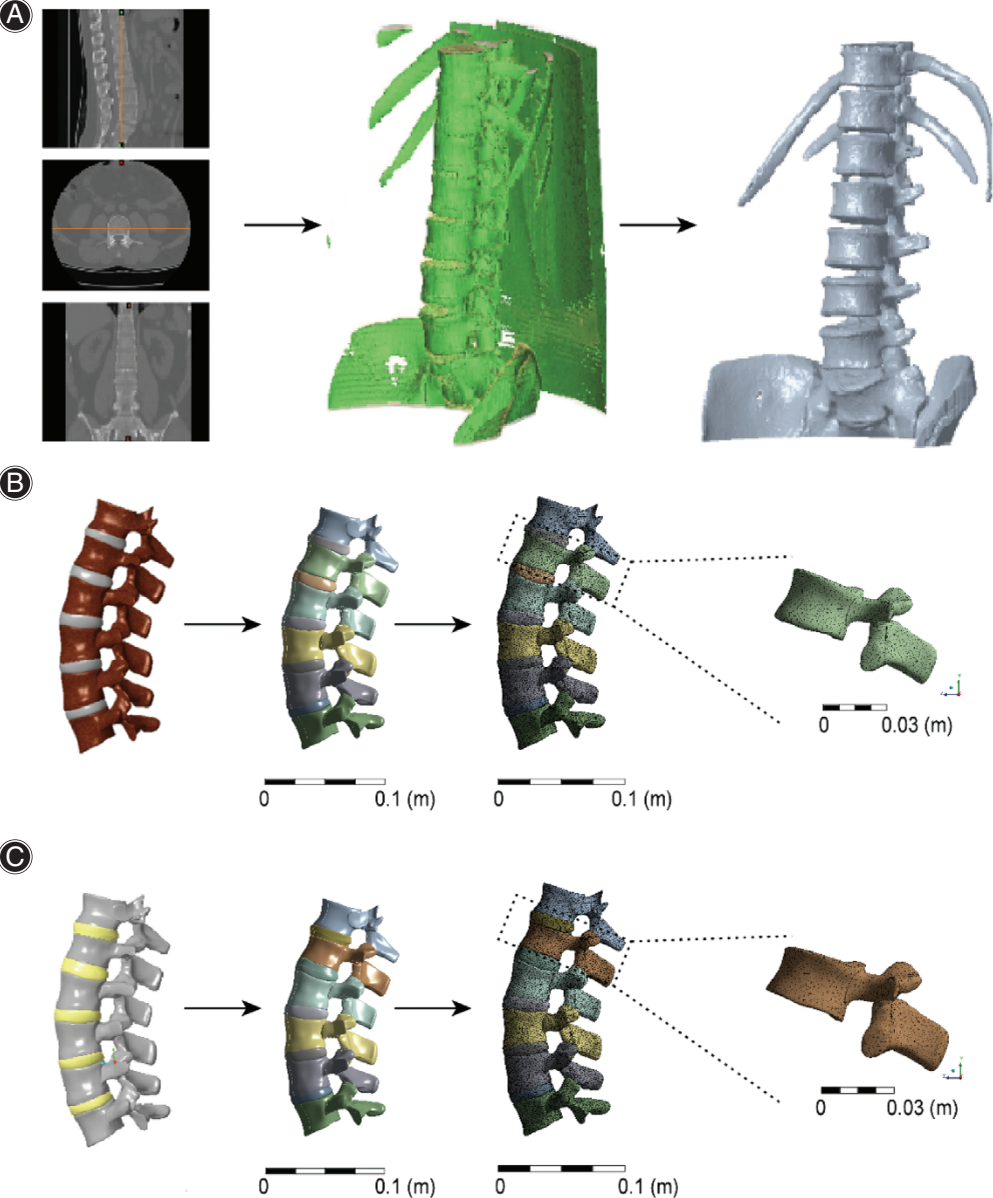
Load application diagram.(A) Working condition 1: in the normal spine model, the center of gravity was shifted toward T12, and a force (Y‐axis) of 200 N and torque of 5.2 Nm (X‐axis) were applied vertically downward toward T12. (B) Working condition 2: in model with a L1 compression fracture, a force (Y‐axis) of 200 N and a torque of 5.2 Nm (X‐axis) were applied vertically downward toward T12. (C) Working condition 3: in the L1 compression fracture model with the spinal protection device, a force (Y‐axis) of 200 N and torque of 5.2 Nm (X‐axis) were applied vertically downward toward T12. The auxiliary device exerted a pre‐tightening force of −60 N (Y‐axis) on the shoulder at a distance of 375 mm from T12. A load of 40 N (Y‐axis) was applied to L1. According to the principle of force translation, the pre‐tightening force on the shoulder was translated to T12, and a force of −60 N was applied to T12 with a torque of 2.25 Nm (X‐axis)

The X–Y plane was used as the horizontal plane. The X–Z plane was used as the coronal plane, and the Y–Z plane was used as the sagittal plane to establish the coordinate system. The six degrees of freedom of the L5 lower vertebral endplate were constrained as boundary conditions. For the purpose of a simple analysis, the weight of the portion of the human body above T12 was set to be 20 kg, and the average distance between the gravity line (GL) and the center of T12 was −26 mm.^15^ Three analysis conditions were set, and the load applied in the three conditions is shown in Fig. 1.

Working condition 1: in the normal spine model, the center of gravity was shifted toward T12 according to the translation principle of forces, and a force (Y‐axis) of 200 N and torque of 5.2 Nm (X‐axis) were applied vertically downward toward T12 (Fig. 1A).

Working condition 2: in model with a L1 compression fracture, the translation principle of forces was the same as that in working condition 1, and a force (Y‐axis) of 200 N and a torque of 5.2 Nm (X‐axis) were applied vertically downward toward T12 (Fig. 1B).

Working condition 3: in the L1 compression fracture model with the spinal protection device, a force (Y‐axis) of 200 N and torque of 5.2 Nm (X‐axis) were applied vertically downward toward T12. The auxiliary device exerted a pre‐tightening force of −60 N (Y‐axis) on the shoulder at a distance of 375 mm from T12. A load of 40 N (Y‐axis) was applied to L1. According to the principle of force translation, the pre‐tightening force on the shoulder was translated to T12, and a force of −60 N was applied to T12 with a torque of 2.25 Nm (X‐axis) (Fig. 1C).

The validity of FEA model was verified in previously published studies.^16^, ^17^ The model parameters in this study were consistent with those published before in the literature.

### Statistical analysis

SPSS20.0 software (SPSS Inc., Chicago, IL, USA) was used for data sorting and processing and statistical analysis, and the measurement data are expressed as $\bar{x}\pm s$. Independent‐samples *T* tests were used for intergroup comparisons. The 0.05 significance level was used, and *p* < 0.05 was considered statistically significant.

## Results

### Successful establishment of 3D finite element model

**Fig. 2:**
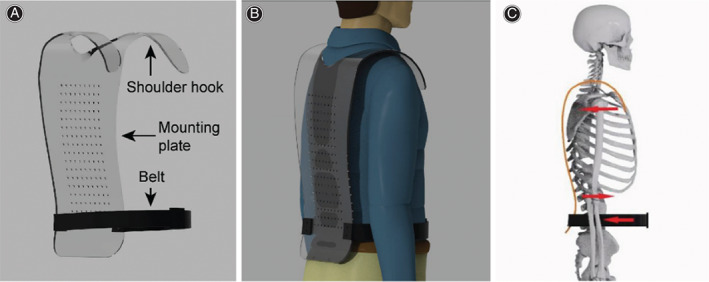
(A) The digital imaging and communications in medicine (DICOM) files were used for 3D reconstruction of the CT images, and then the 3D reconstruction model was transformed into STL files. (B) After the meshes and joints of the normal lumbar spine were set, solid modeling was conducted, and the ligaments were connected. (C) After the meshes and joints of the lumbar spine were set for L1 fractures, solid modeling was performed, and ligamentous connections (L1 anterior margin compressed by 20%) were made

The CT images in the DICOM format were imported into Mimics Medical 21 (Materialise, Michigan, USA). Next, 3D reconstruction was performed, and the model was converted into the STL file format for output (Fig. 2A). The STL data were then imported into Creo Parametric software (PTC, Massachusetts, USA). A solid model (including thoracic 12 vertebrae) was built through meshes, and the lumbar vertebrae were connected with normal lumbar (Fig. 2B) and L1 (Fig. 2C) compression fractures. The files were saved as IGS files and imported into ANSYS Workbench 2020R1 software (ANSYS, Pennsylvania, USA) for finite element analysis.

To simplify the model, the spine and intervertebral discs were analyzed as entities, the mesh size was set to be 2 mm to automatically divide the mesh, and the ligaments were simplified by spring element connections. The finite element parameters of all major materials related to the model were set according to the definition of osteoporosis, cortical bone, and endplate. The posterior elements of the elastic modulus were 67% of the normal, the elastic modulus of the cancellous bone was 34% of the normal, and that of the soft tissue structure remained the same, as shown in Table 1.^18^, ^19^, ^20^, ^21^, ^22^ The materials were defined as linear elastic and isotropic materials. Among them, the cortical bone, cancellous bone, endplate, posterior element of the vertebral body and nucleus pulposus were defined as solid units of linear isotropic elastic materials, while the units of the anterior longitudinal ligament, posterior longitudinal ligament, transverse intervertebral ligament, interspinous ligament, supraspinous ligament and yellow ligament were defined as nonlinear elastic units.

**TABLE 1:** Parameters of the finite element model of human thoracic‐lumbar vertebrae

Component	Young modulus (MPa)	Stiffness coefficient	Poisson's ratio
Cortical bone	8040 (67% “normal,” 12,000)	0.3	
Vertebral bony endplate	2680 (67% “normal,” 4000)	0.4	
Posterior elements	2345 (67% “normal,” 3500)	0.25	
Cancellous bone	34 (34% “normal,” 100)	0.25	
Intervertebral disc	295	0.35	
ALL	–	60,000	–
PLL	–	50,000	–
ITL	–	40,000	–
ISL	–	40,000	–
SSL	–	20,000	–
LF	–	160,000	–

Abbreviations: ALL, anterior longitudinal ligament; PLL, posterior longitudinal ligament; ITL, inter transverse ligament; ISL, interspinous ligament; SSL, supraspinous ligament; LF, ligamentum flavum.

### Establishment of a finite element model for the spinal protection device

**Fig. 3:**
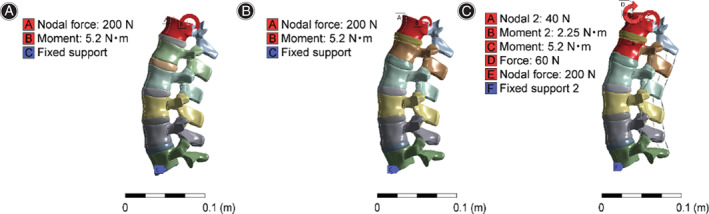
(A) Spinal protection device. (B) Simulation diagram of a person wearing the device. (C) Diagram of the spinal protection device forces

For this new spinal protection device, the upper part is a shoulder hook integrated with a spinal support plate, and the lower part is connected to a belt (Figure 3A). When the shoulder hook is placed on the shoulder and the belt is tightened (Fig. 3B), a backward force is applied to the shoulder and abdomen, and a forward force is applied around loin 1 because the middle of the spine support plate protrudes forward (Fig. 3C). Osteoporotic thoracolumbar compression fractures lead to anterior vertebral collapse and a forward‐directed center of gravity. On the basis of the characteristics of this disease, this spinal protection device was invented to reduce the stress on the anterior vertebral edge and direct the center of gravity posteriorly. Finally, finite element software, ANSYS Workbench 2020R1 (ANSYS, USA), was used for following finite element analysis.

### Distribution characteristics of the L2 maximum stress in different States

**Fig. 4:**
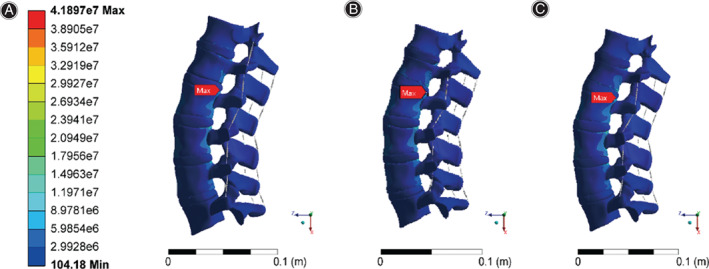
(A) Stress distribution for working condition 1. (B) Stress distribution for working condition 2. (C) Stress distribution for working condition 3

Through finite element analysis, the equivalent stress distribution under three working conditions is obtained, as shown in Figure 4.The maximum stress of working condition 1 was 41.9 MPa, that of working condition 2 was 37.8 MPa, and that of working condition 3 was 32.7 MPa. The maximum stress points were all located on lumbar vertebrae 2 (the position of the red arrow in Fig. 4). Overall, according to the comparison of condition 1 and condition 2, when the fracture deformation occurred, the overall stress distribution moved forward, and the maximum stress value decreased by 9% after the fracture. The mean overall stress values were 0.923 and 0.954 MPa for condition 1 and 2, respectively, with a 3% increase after the fracture.

**Fig. 5:**
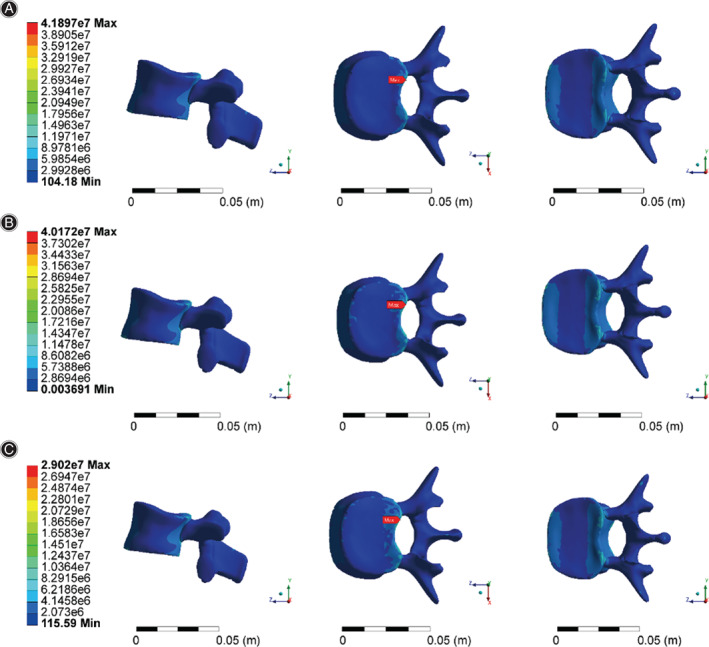
(A) Stress distribution of the lumbar 2 vertebral body in working condition 1. (B) Stress distribution of the lumbar 2 vertebral body in working condition 2. (C) Stress distribution of the lumbar 2 vertebral body in working condition 3

The maximum stress point was extracted from the L2 vertebral body (Fig. 5). The stress distribution of the fractured vertebral body changed in accordance with the overall stress moving forward, and the maximum stress point was located in the posterior region of the L2 upper endplate under three working conditions. Compared with working condition 2 (37.8 MPa), working condition 3 (32.7 MPa) exhibited a lower maximum stress value by 13%.

### Distribution characteristics of the L1 maximum stress in different states

**Fig. 6:**
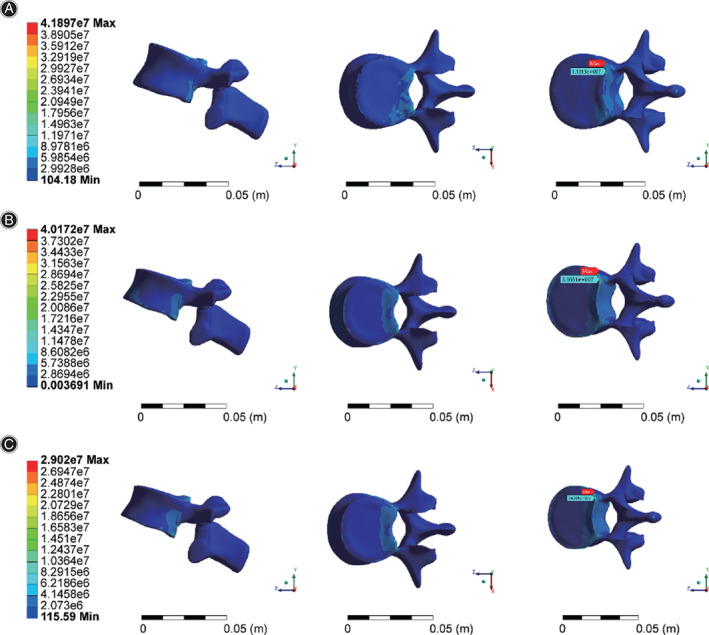
(A) Stress distribution of the lumbar 1 vertebral body in working condition 1. (B) Stress distribution of the lumbar 1 vertebral body in working condition 2. (C) Stress distribution of the lumbar 1 vertebral body in working condition 3

Under three working conditions, the position of maximum stress on L1 appeared at the posterior edge of the lower endplate (Fig. 6), and the values were 10.4 MPa (working condition 1), 33.6 MPa (working condition 2) and 27.1 MPa (working condition 3). The maximum stress of the L1 vertebral body before and after the fracture was compared. It increased by 3‐fold after the fracture, and the stress produced by all parts of the vertebral body significantly increased. The maximum stress on the L1 vertebral body decreased by 20% after the auxiliary device was applied.

### Variation characteristics and statistical analysis

**Fig. 7:**
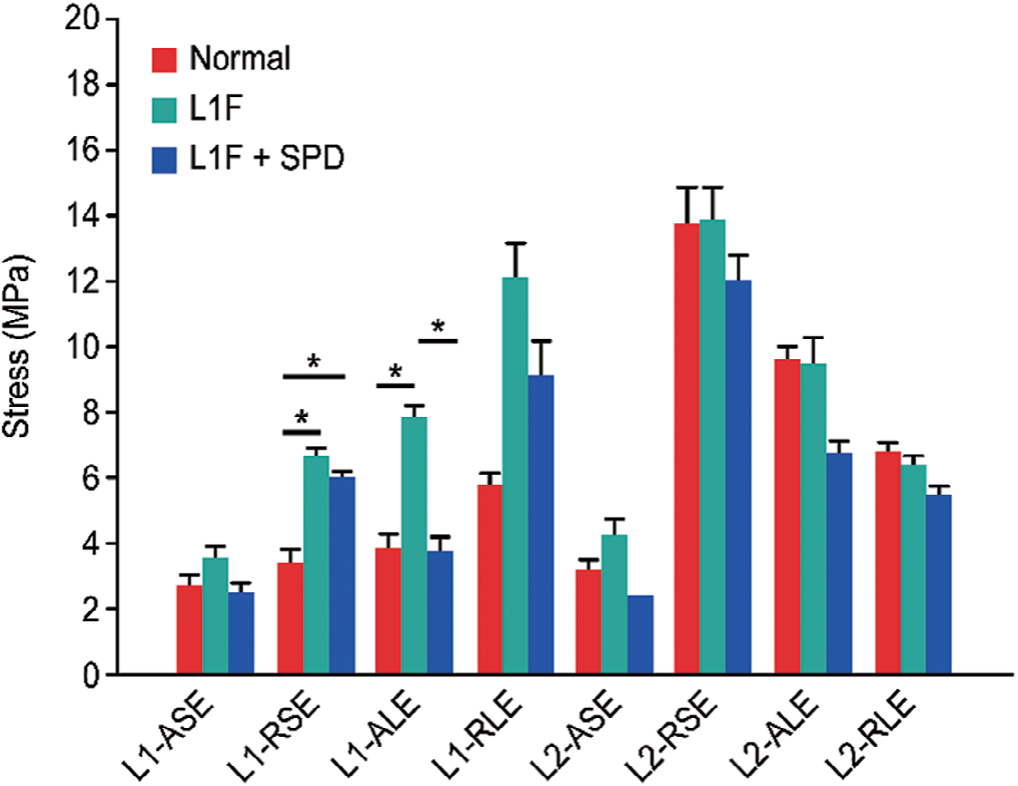
Comparison of the average stress at the anterior and posterior points of the upper and lower endplates of the L1 and L2 vertebrae under three working conditions. The changes in stress distribution are shown at each position under the normal state, the lumbar 1 vertebral fracture and under the protection of the new spinal protection device (* *P* < 0.05). After L1 fracture, the stress at the upper and lower endplates of L1 and the upper endplates of L2 increased, and the increase in stress at the upper and lower endplates of L1 was significant (*P* < 0.05). After the new type of spinal fixation device was applied, the stress at the L1 and L2 vertebral endplates decreased to a certain extent, especially that at the L1 vertebral body (*P* < 0.05)

The lumbar 1 and lumbar 2 vertebrae were selected according to the stress neutron diagram characteristics, and the anterior and posterior parts of the upper and lower endplates of each vertebra were selected. A total of five points at each location were measured, and the average value was taken for comparison and statistical analysis (Tables 2, 3, 4 and Fig. 7). The stress at the upper and lower endplates of L1 and the upper endplates of L2 increased after the fracture, and the increase in stress at the rear of the superior endplate of L1 (*t* = −3.307, *p* < 0.05) and the anterior inferior vertebral endplate of L1 (*t* = −2.751, *p* < 0.05) was significant. After the new type of spinal fixation device was applied, the stress at the L1 and L2 vertebral endplates decreased to a certain extent, especially that at the L1 vertebral body (*t* = −2.437, *p* < 0.05).

**TABLE 2:** Comparison of stress values between normal state and vertebral fracture state

Position	Normal state (MPa)	Fracture state (MPa)	*T* value	*P* value
L1‐ASE	2.65 ± 0.95	3.54 ± 1.24	−1.279	0.237
L1‐RSE	3.38 ± 1.47	6.61 ± 1.61	−3.307	0.011
L1‐ALE	3.83 ± 1.61	7.80 ± 2.80	−2.751	0.025
L1‐RLE	5.75 ± 2.13	12.06 ± 12.76	−1.091	0.307
L2‐ASE	3.18 ± 0.98	4.19 ± 2.16	−0.955	0.368
L2‐RSE	13.71 ± 15.34	13.82 ± 13.72	−0.013	0.990
L2‐ALE	9.57 ± 3.97	9.48 ± 7.72	0.023	0.982
L2‐RLE	6.76 ± 1.91	6.37 ± 1.60	0.351	0.735

Abbreviations: ASE, anterior superior endplate of vertebral body; ALE, anterior inferior vertebral endplate; RLE, the rear of the inferior vertebral endplate; RSE, the rear of the superior endplate.

**TABLE 3:** Comparison of stress values between normal state and SPD state

Position	Normal state (MPa)	SPD state (MPa)	*T* value	*P* value
L1‐ASE	2.65 ± 0.95	3.15 ± 0.66	−0.963	0.364
L1‐RSE	3.38 ± 1.47	5.62 ± 1.42	−2.452	0.040
L1‐ALE	3.83 ± 1.61	6.10 ± 2.71	−1.609	0.146
L1‐RLE	5.75 ± 2.13	10.17 ± 10.81	−0.898	0.395
L2‐ASE	3.18 ± 0.98	2.81 ± 0.24	0.812	0.440
L2‐RSE	13.71 ± 15.34	12.70 ± 10.50	0.121	0.906
L2‐ALE	9.57 ± 3.97	8.39 ± 3.97	0.473	0.649
L2‐RLE	6.76 ± 1.91	5.14 ± 0.81	1.748	0.136

Abbreviations: ASE, anterior superior endplate of vertebral body; ALE, anterior inferior vertebral endplate; RLE, the rear of the inferior vertebral endplate; RSE, the rear of the superior endplate; SPD, spinal protection device.

**TABLE 4:** Comparison of stress values between vertebral fracture state and SPD state

Position	Fracture state (MPa)	SPD state (MPa)	*T* value	*P* value
L1‐ASE	3.54 ± 1.24	3.15 ± 0.66	0.626	0.549
L1‐RSE	6.61 ± 1.61	5.62 ± 1.42	1.025	0.335
L1‐ALE	7.80 ± 2.80	3.72 ± 0.46	−2.437	0.027
L1‐RLE	12.06 ± 12.76	10.17 ± 10.81	0.252	0.807
L2‐ASE	4.19 ± 2.16	2.81 ± 0.24	1.421	0.227
L2‐RSE	13.82 ± 13.72	12.7 ± 10.50	0.145	0.888
L2‐ALE	9.48 ± 7.72	8.39 ± 3.97	0.282	0.785
L2‐RLE	6.37 ± 1.60	5.14 ± 0.81	1.537	0.163

Abbreviations: ASE, anterior superior endplate of vertebral body; ALE, anterior inferior vertebral endplate; RLE, the rear of the inferior vertebral endplate; RSE, the rear of the superior endplate; SPD, spinal protection device.

## Discussion

In this study, to facilitate the conservative prevention and treatment of osteoporotic vertebral compression fractures, a new type of spinal protection device was invented according to the biomechanical characteristics of the spine to effectively correct the center of gravity from being directed forward after vertebral fractures. The new spinal protection device is simple in design and easy to wear. With this device, it is not necessary to stay in bed for a long time for OVCF patients. Under the protection of the new spinal protection device, patients can stand or walk. Through finite element analysis, it was found that after the spinal protection device was used, the stress in the anterior part of the L1 vertebra was significantly lower than that after the fracture, even lower than that of a normal vertebra, which indicated that the new spinal protection device can effectively reduce the magnitude of stress in the anterior part of the fractured vertebra.

### Stress distribution characteristics of L1 vertebral body

At the level of the L1 vertebra, the normal human center of gravity is located 17 mm in front of the center of the L1 vertebra. With muscle and ligament contractile forces being directed posteriorly and the center of gravity being directed toward the front of the vertebra, a mechanical balance with the vertebra serving as a fulcrum is constructed. Under normal conditions, both ends are in equilibrium. When the anterior edge of the vertebral body is fractured, kyphosis occurs, and the body's center of gravity moves forward, which is bound to increase the moment arm of the center of gravity from the fulcrum, increasing the risk of fracture caused by stress being concentrated on the anterior edge of the vertebral body. Dall'Ara et al.^23^ established a finite element model of the thoracolumbar segment of the vertebral body, conducted an axial loading experiment, and simulated the mechanism of thoracolumbar injury. They found wedge compression fractures occurred first at the anterior edge of the vertebrae under the axial load of the thoracolumbar spine, which was consistent with our results and supported our study.

### Designing a new type of spinal protection device

In this study, it was found that an anterior fracture of the upper and lower endplates of the L1 vertebra resulted in an obvious increase in stress after collapse, while the stress decreased significantly after the application of the spinal protection device. Moreover, the change in stress at the anterior lower endplate of L1 was statistically significant. According to these, we designed a new type of spinal protection device. The new type of spinal protection device counter balances spines with kyphosis deformities after fractures, so that the center of gravity moves backward, effectively reducing the stress distributed in the anterior and middle columns of the vertebral body, maintaining the spinal mechanical balance, stabilizing the vertebral body, reducing pain, and preventing the occurrence of new compression fractures. The spinal protector effectively restricts the forward bending of the trunk and prevents the forward movement of the body's center of gravity during falls, which helps individuals maintain balance. The literature has suggested that abnormal activity of the periosteum and vertebral endplate, microfractures of the vertebral body and micromovements of the fracture area stimulate nerve endings in the vertebral body, which is one of the main causes of pain.^24^ Because the new spinal protection stabilizes the spine and reduces abnormal movement at the fracture site, it reduces the severity of local pain.

### Prevention and treatment effects of the spinal protection device

By building a 3D finite element model of a human lumbar spine with a fracture and applying the forces generated by the new spinal protection device, we analyzed the size, distribution and variation characteristics of the stress on the lumbar spine under the vertical impact load by simulation, explained the mechanism of human lumbar fracture from a biomechanical point of view, and demonstrated the effectiveness and practicability of the new spinal protection device. The finite element study has confirmed that stress is concentrated in the fractured region of the vertebral body,^25^ and it was found that the fracture region is mainly distributed in the upper and central parts of the vertebral body, which indicate that the stress of the fractured vertebral body is usually concentrated in the upper and middle parts of the anterior and middle columns of the vertebral body under axial loads. This new type of spinal protection device can support the arch of the spine, can reduce and disperse the harmful stress in the thoracolumbar segment of the spine, and at the same time, make the area of stress move toward the middle and posterior columns, thereby effectively fixing and protecting the thoracolumbar segment. For patients with visual analogue scale (VAS) pain scores below 6 points, treatment with the new type of spinal protection device is the first choice, as it can effectively reduce the stress distributed in the anterior and middle columns of the vertebral body, prevent kyphosis from gradually progressing, and effectively protect the thoracolumbar segment.

However, some limitations of this study should be addressed further. First, many individuals who have a history of OVCFs are prone to falls due to age‐related physiological dysfunction and weakness, which are often neglected.^26^ The posture of the body during a fall and the strength of the relevant muscles is different across individuals, so it is difficult to simulate falls. The FEA method needs to be studied further for research directions such as falls. Second, as the model includes electronic data with infinite repeatability, it can perform repeated operations for a specific intervention factor or different intervention factors to perform relevant analysis. However, as FEA is a numerical simulation method, clinical observations and postoperative follow‐ups are needed to obtain comprehensive and accurate reference data and theoretical guidance for the occurrence, development and treatment of spinal fractures. Lastly, a large number of patients is needed to validate the effects of this device in conservative treatments of OVCF patients.

### Conclusions

The finite element analysis results of the prevention and treatment effects of the new spinal protection device on OVCFs show that the new spinal protection device can change the stress distribution of the spine and is effective in the prevention and treatment of OVCFs, and has a wide application prospect. For patients with a VAS score below 6, the new spinal protective device may become the preferred treatment.
